# Grounding Word Learning in Space

**DOI:** 10.1371/journal.pone.0028095

**Published:** 2011-12-14

**Authors:** Larissa K. Samuelson, Linda B. Smith, Lynn K. Perry, John P. Spencer

**Affiliations:** 1 Department of Psychology and Delta Center, University of Iowa, Iowa City, Iowa, United States of America; 2 Department of Psychological and Brain Sciences, Indiana University, Bloomington, Indiana, United States of America; University of Queensland, Australia

## Abstract

Humans and objects, and thus social interactions about objects, exist within space. Words direct listeners' attention to specific regions of space. Thus, a strong correspondence exists between where one looks, one's bodily orientation, and what one sees. This leads to further correspondence with what one remembers. Here, we present data suggesting that children use associations between space and objects and space and words to link words and objects—space binds labels to their referents. We tested this claim in four experiments, showing that the spatial consistency of where objects are presented affects children's word learning. Next, we demonstrate that a process model that grounds word learning in the known neural dynamics of spatial attention, spatial memory, and associative learning can capture the suite of results reported here. This model also predicts that space is special, a prediction supported in a fifth experiment that shows children do not use color as a cue to bind words and objects. In a final experiment, we ask whether spatial consistency affects word learning in naturalistic word learning contexts. Children of parents who spontaneously keep objects in a consistent spatial location during naming interactions learn words more effectively. Together, the model and data show that space is a powerful tool that can effectively ground word learning in social contexts.

## Introduction

Language is fundamentally a social phenomenon and social cues—following the eye gaze, point, or gesture of a speaker—are highly informative about the speaker's state of mind and the intended referent of a spoken word [1]–[4]. By some accounts, this ability to infer the internal mental state of others from such actions, a competence sometimes called “mindreading,” is what distinguishes human language from animal communication [2]–[3]. Relevant to this idea, human infants have been repeatedly shown to be very good at reading these social cues to intended referents [1], [5]. We know little, however, about the mechanisms on which these abilities rest. Here, we pursue the hypothesis that the spatial dimensions of speakers' actions engage spatially organized processes of attention and working memory *in the listener*. Social cues—eye gaze, points, and gestures—are spatial and orient the listener's visual attention. The orientation of visual attention is known to play an essential role in the working memory processes that bind features (including multimodal features, [5]) into unified representations [6]. In brief, toddlers and parents may not be able to literally share minds, but they have similar cognitive machinery that allows them to do the next best thing—share a common space [7], [8].

Experiments 1 through 4 are modeled on Baldwin's classic demonstration of young children's ability to read referential intent [1]. These studies document the role of spatially-grounded processes in children's mapping of a name to a referent. A dynamic neural field (DNF) model that provides a process account of how children bind visual features to names by virtue of a shared space, successfully simulates children's performance and makes a new prediction tested in Experiment 5. Finally, Experiment 6 connects these cognitive processes to everyday social word learning.

## Results

### Behavioral Experiments

**Figure 1:**
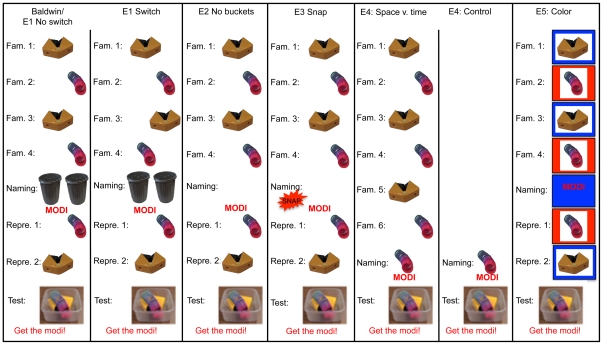
The tasks used in Experiments 1–5. The far left panel shows the original Baldwin (1993) task [1], a classic demonstration of children's use of social cues in word learning, that we used as the basis of our studies. Experiment 1 replicates the Baldwin task in the No-Switch condition and tests the necessity of spatial consistency for children's performance in the Switch condition. Experiment 2 tested the necessity of the buckets in this task by removing them from the critical naming event—the experimenter simply pointed to the empty space on the table where one of the objects had been during familiarization and said the name. In Experiment 3 a more diffuse spatial cue was provided. During the naming event the experimenter held her hand out to one side of the table and snapped, directing children's attention, generally, to one side or the other. Experiment 4 pitted prior consistency in space against temporal contiguity. We gave children consistent spatial experience with two objects as in the previous experiments, but during the naming event in the experimental condition the experimenter pointed to and labeled a visibly-present object. Critically, the object was in an inconsistent spatial position. A control condition confirmed that children this age would bind a name and object presented ostensively. Experiment 5 tests the prediction from the DNF model that children cannot use color cues to bind names with objects. During familiarization, each object was presented at the center of the table but consistently on either a red or a blue tray. During the naming event, one of the two colored trays was presented at the center of the table and the experimenter pointed to it and said the label. The final test event was exactly as in the prior experiments.

The left panel in Fig. 1 shows Baldwin's original task. A novel object is presented to a child on one side of a table (see “Fam. 1”). This object is removed and a second novel object is presented on the other side of the table (“Fam. 2”). This is repeated across several familiarization trials. Then, out of view of the child, each object is placed in a separate opaque bucket. The buckets are placed on either side of the table. The experimenter looks into one bucket and says “Modi!” The object from the other bucket is then taken out and placed on its side of the table (“Repre. 1”). After the child examines it, it is removed and the other object is placed on its side of the table (“Repre. 2”). After examination, it is also removed. Both objects are then placed in a transparent container at the center of the table. The container is pushed toward the child, and the experimenter asks, “Can you get me the modi?” Baldwin [1] reported that children robustly retrieved the object that had been in the attended bucket when the name was provided, supporting the claim that word learning depends on the inferred referential intent of the speaker and not the temporal synchrony of a heard word and a seen object.

But does children's performance in this task depend on the *spatial* coincidence of attention when the object is seen and when the novel word is heard? In the task, children look to and reach for each object on different sides of the table during familiarization. During naming, they look at a bucket on one side, in a direction that overlaps with the spatial experience of one object. Prior research suggests that both adults [9] and young infants [10] can associate semantic information with locations in space and maintain indices of this spatial information over time. Thus the first four experiments presented here test whether the spatial coincidence of attention and objects at the point of naming is critical to children's ability to bind names to unseen objects.

In Experiment 1, 16–18 month-old children participated in either a No-Switch (original Baldwin) or a Switch condition. The Switch condition (Fig. 1) differed from Baldwin's procedure in that the right-left placement of the objects was switched on the third familiarization trial. At test, children were given the spoken name and asked to select the referent from a centrally presented container. If in the original Baldwin condition children had succeeded because they had read the referential intent of the experimenter, they should perform well in both the No-Switch and Switch conditions here, because the referential cues in both conditions were strong and identical. If, however, the spatial alignment of the object-familiarization and naming events were critical to *binding* the name and referent, children in the No-Switch condition should still map the name to the object in the bucket. By contrast, children in the Switch condition should guess randomly because neither object has a consistent spatial mapping.

**Figure 2:**
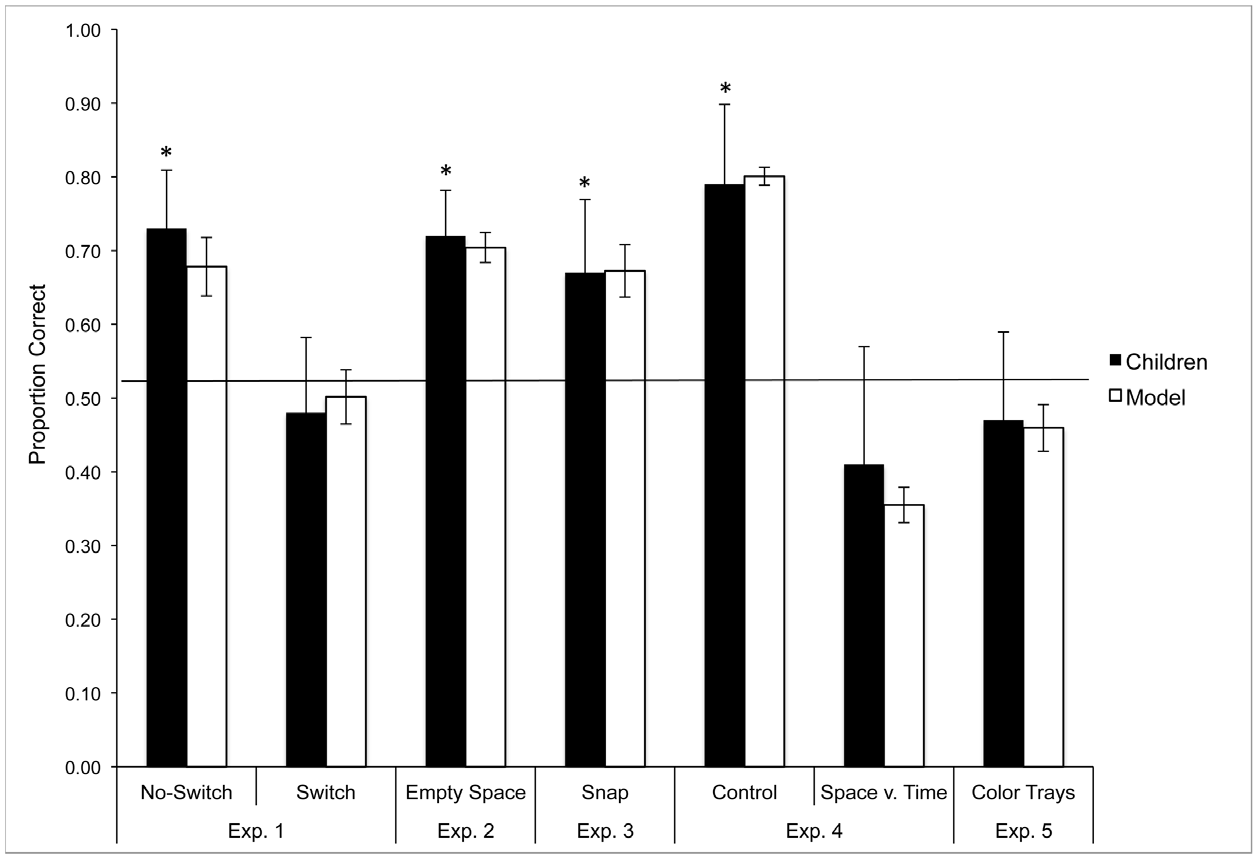
Performance of children and model in Experiments 1–5. Children's percent of correct choices for each experiment (black bars) with standard deviations (range of error bars). *s indicate performance significantly above chance (.50 in a two item forced-choice task). The mean performance of the Dynamic Neural Field model (across 12 batches of simulations) for all experiments is also shown (white bars). Error bars show the standard deviation of the model's performance (across 12 batches of simulations) per condition, relative to the target means.

The results (Fig. 2) support the latter prediction and show that spatial consistency is necessary for children to map the name to the hidden object and that the clear referential intent of the experimenter is not sufficient. Children in the No-Switch condition who saw the objects consistently on a particular side of the table during familiarization chose the intended referent on .73 of test trials, which is reliably greater than chance (.50), *t*(11) = 5.01, *p*<.01, and comparable to Baldwin's original finding. But when the spatial location of the objects varied prior to naming, children's choices did not differ from chance levels (.48, *t*<1.00), but did differ significantly from performance in the No-Switch condition, *t*(22) = 3.34, *p*<.01. One concern with young children is whether they understood the task in both conditions. Thus, we included ‘filler’ test trials in which children were asked to pick one of two familiar objects by name (“where's the spoon?”). Children in all conditions (in experiments 1–5) chose the named toy on more than .80 of the filler trials showing that they understood the testing procedure.

Results of Experiment 1 suggest that spatial consistency is a key factor for success in the Baldwin task and that the clear referential intent of the speaker is not sufficient. Still, clear referential intent may be necessary. To probe this issue, we took away the buckets in Experiment 2. Naming objects unseen because they are in buckets is quite natural within a social pragmatics account. Providing a name while pointing to an empty table-top would seem a more unusual event from a social pragmatics perspective. Accordingly, during naming in Experiment 2, the experimenter pointed to the empty space on the table where one of the objects had been during familiarization and said the name. Children picked the target object on .72 of the test trials (Fig. 2), which is significantly different from chance, *t*(15) = 7.0, *p*<.001. Thus, spatial consistency is sufficient for children to succeed even given what is likely an unusual naming event and an unusual social pragmatics context for children this age (see [11], and [12] for evidence that parents of children in the age range tested here do not often talk about absent objects).

Experiment 3 took this one step further. At the moment the name was provided, with no objects in view, the experimenter held her hand out to one side of the table, snapped her fingers in the air, and said the name (“modi”), pulling the child's visual attention in one direction at the moment of naming. If the direction of visual attention plays a role in binding the name to the previously-seen object, then even this diffuse spatial cue should be sufficient for children to map the name to a spatially-anchored memory of an object. Children chose correctly on .67 of test trails, significantly above chance levels, *t*(15) = 3.47, *p* = .01. Experiments 1–3, therefore, show that children's ability to map a name to an object that is not physically present depends critically on the direction of visual attention at the moment of naming and on object memories tied to, or indexed by, locations in the task space (see [10] for related data with 6 month-old infants).

The original Baldwin task was designed to create an ambiguous word-learning context in which children relied on social pragmatics. Everyday word learning, of course, often has less ambiguity in that children hear a name when they are looking at an object with a speaker giving clear referential cues; thus, the seen object, the heard name, and the referential cues are spatially and temporally aligned. Experiment 4 asks whether it is sufficient for these cues to align in-the-moment, or whether spatially anchored object memories still play a role in these unambiguous naming contexts. Children were given consistent spatial experience with two objects prior to naming and then an unambiguous naming event in which the experimenter pointed to and labeled a visibly-present object *at a location incongruent with the previous experiences* (Fig. 1). To measure the role of the spatially incongruent experiences prior to naming, this was compared to a control condition in which the experimenter again pointed to the object while naming but with no prior object familiarization. Consistent with other findings (e.g., [13]) young children selected the named object at test .79 of the time in the Control condition, which is different from chance levels, *t*(13) = 4.95, *p*<.001, (Fig. 2). In the Experimental condition, however, the inconsistent spatial experience disrupted word learning: they selected the ostensively named object only .41 of the time, a result that is not different from chance, *t*(15) = −1.05, *ns*.

### Model

Although the social pragmatics perspective has generated a wealth of empirical data on young children's “mindreading,” it is not clear what mechanisms underlie such abilities. Researchers often point toward the mirror neuron system as a possible neural basis ([14], and see [15] for discussion) however, the behaviors captured by the mirror neuron system are quite different from the behaviors probed in developmental studies, and there is much debate regarding the mirror neuron system's role in “mindreading” [15]. Thus, at present, there is no neurally-grounded framework that captures how children behave in experiments like the Baldwin experiment that are purported to tap into children's understanding of social pragmatics.

One way in which the shared-space account goes beyond a social pragmatics perspective is its ability to tie word learning to known neural processes of spatial attention, spatial memory, and associative learning. The dynamic neural field (DNF) model presented here is a demonstration proof that such links can be formalized in a way that quantitatively captures children's performance and generates novel behavioral predictions open to empirical test (see Experiment 5). The DNF model is based on an understanding of the neural population dynamics that underlie elementary forms of perception [16], working memory [17], [18], and motor planning [19], as well as the development of these cognitive processes [20], [21]. As such, the model provides a theoretical bridge between word learning in a shared space and other cognitive abilities that emerge in early development.

The DNF model also provides solutions to several theoretical challenges evident in the Baldwin task. First, word learning is often treated as a computationally slow process requiring many presentations [22]. Likewise, visual feature extraction for novel objects is often modeled in a way that requires many exposures to an object in different poses [23]. This contrasts with children's ability in the present experiments to pick out the referent of the novel word after a single naming event. The DNF model we propose achieves such “fast-mapping” using an architecture recently proposed by Faubel and Schöner [24]. This architecture uses previously-learned mappings from low-level visual cues to cortical neural populations with receptive fields “tuned” to features such as hue, aspect ratio, and orientation. These features are ‘bound’ on-the-fly using words, and associations are built over a trial-to-trial timescale using a variant of Hebbian learning.

Next, we incorporate a second architecture proposed by Johnson and colleagues [25] that provides a solution to a different challenge—binding visual features together into an integrated object representation. As one progresses through the ventral object recognition pathway of the primate visual system, there are several key changes in neural response properties, including changes in the complexity of the features coded [26] and a dramatic increase in receptive field sizes and an accompanying decrease in the spatial resolution of receptive fields [27], [28]. This type of neural coding can create a binding problem in vision: it can be difficult to know which features go together when two or more objects are presented simultaneously ([29], [30]; although see [31] for critiques of this view). Johnson et al. [25] implemented a neurally-grounded approach to this problem by binding features together by virtue of their shared spatial location using detailed spatial information represented in cortical fields in the dorsal visual pathway (for related ideas, see [32]). We integrate this architecture into the DNF model used here. This enables our system to bind visual features together in a spatial frame of reference and, in concert with the Faubel and Schöner architecture, to ground word learning in space and time.

**Figure 3:**
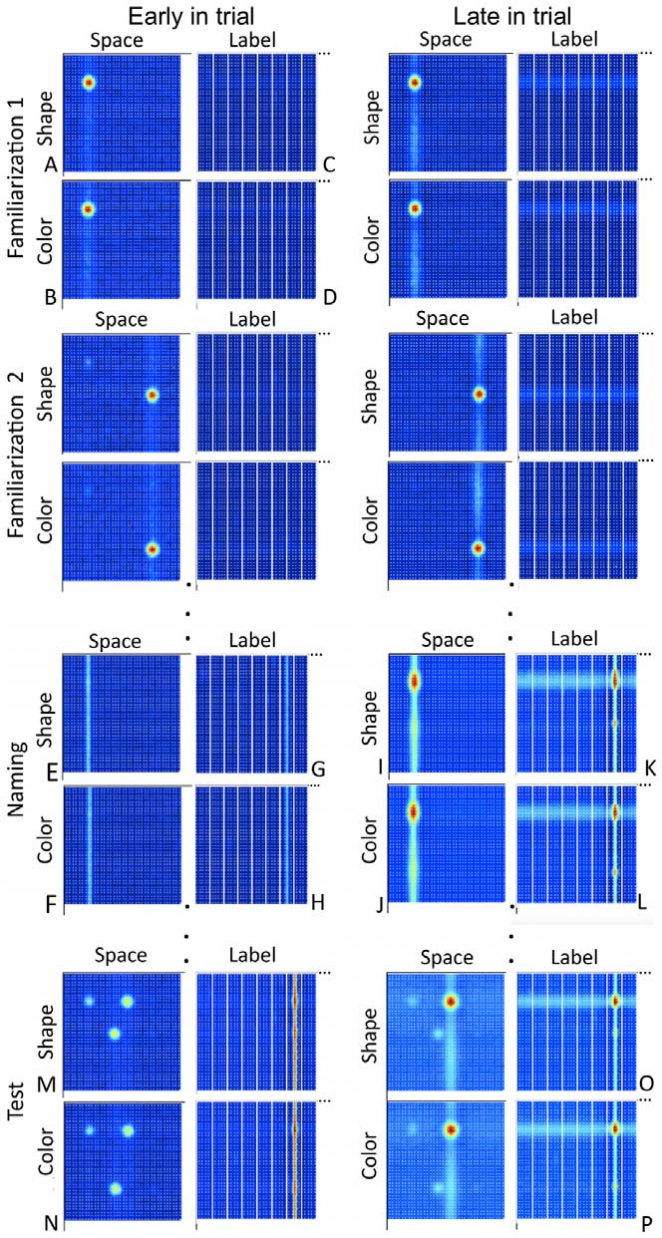
Dynamic Neural Field (DNF) model that captured Experiments 1–4 performance and predicted Experiment 5 behavior. Panels A and B show a variant of Johnson et al.'s [10] model of visual feature binding; panels C and D show a variant of the Faubel and Schöner's [17] model of fast object recognition. Our integration brings these prior models together to encode and bind visual features in real time as “peaks” of neural activation built in the shape-space (red hot spots in A) and color-space fields (red hot spots in B) via local excitation and surround inhibition (see [38]). Binding is achieved through the shared spatial coupling between these fields. Labels (words) are fed into the label-feature fields shown in C and D. These fields can bind labels to the visual features encoded by the visuo-spatial system via in-the-moment coupling across the shared feature dimensions (shape to shape; color to color). A Hebbian process enables the model to learn which features were where from trial to trial and also learn the label-feature associations quickly to influence performance on subsequent test trials. This figure also shows a simulation of the model at key points in time as we capture events in our experimental task.

Figure 3 shows the new DNF model we propose that builds on these previous innovations and uses a table-centered frame of reference to link both actors (see [33]). Panels A and B of Figure 3 show a variant of the Johnson et al. model with two cortical fields—a shape-space field and a color-space field. These cortical layers have cells with bimodal receptive fields: they are sensitive to, for instance, both the shape of an object (e.g., its aspect ratio) and its spatial position. Note that Johnson et al. demonstrated that localized “peaks” of activation (see red circles of activation in Fig. 3A) can build in such cortical fields via local excitation and surround inhibition even with broad spatial receptive fields, thereby mimicking the tuning properties of neurons in the ventral pathway [27], [28]. These localized peaks—“bound” object representations in the model—are achieved through coupled activation along the shared spatial dimension which is evident in Figure 3A and 3B: there is a light vertical “ridge” of activity passed between the shape-space and color-space fields on the left side of these panels—the model has encoded the shape and color of the object on the left. In the present report, we added a Hebbian learning process to the feature-space fields [34]. Consequently, the model learns which features were where from trial to trial.

Panels C and D of Figure 3 show the second part of the DNF architecture—a variant of the Faubel and Schöner model with two label-feature fields. Once again, these cortical layers have cells with bimodal receptive fields. Now, however, they are receptive to input from label neurons—population representations of the spoken word—and featural input from the feature-space fields (A, B). These fields, therefore, bind labels to the visual features encoded by the visuo-spatial system via in-the-moment coupling across the shared feature dimensions (shape to shape; color to color). As in Faubel and Schöner [24], a Hebbian process enables the model to learn these label-feature associations quickly and influence performance on subsequent test trials. Note that neural interactions across the label dimension are winner-take-all with sharp boundaries between one label and the next (i.e., local excitation spreads minimally from one unit to the next; see σ*_e,z_* in Table 1). Such interactions thus capture the discrete-like nature of word representations common in connectionist models of early word learning (e.g., [22], [35], [36]).

**Table 1:** Parameter values for DNF model.

Type	general	self-excitation_x,z_	surround inhibition_x,z_	self-excitation_y_	surround inhibition_y_
Global					
	*τ_e_* = 80				
	*τ_i_* = 10				
	β_within-field_ = 5				
	β_between-fields_ = 1				
	h_test_ = −.1				
	h_q_ = .5				
	σ_noise_ = 1				
	*q* = .075				
	field size = 91				
Space-Feature Fields					
	*h* = −6.35	*c_e,x_* = .8	*c_i,x_* = .3	*c_e,y_* = .8	*c_i,y_* = .3
	*k_i,x_* = .18	*σ_e,x_* = 3	σ_i,x_ = 18	*σ_e,y_* = 3	σ_i,y_ = 6
Label-Feature Fields					
	*h* = −11.55	*c_e,z_* = 1.6	*c_i,z_* = .3	*c_e,y_* = 1.6	*c_i,y_* = .6
	*k_i,z_* = .35	*σ_e,z_* = 1	σ_i,z_ = 60	*σ_e,y_* = 3	σ_i,y_ = 6
Gaussian Projections Across Space					
	*c_e_* = .162				
	*σ_e_* = 3				
Gaussian Projections Across Labels					
	*c_e_* = .4				
	*σ_e_* = 1				
Gaussian Projections Across Features					
	*c_e,z→x_* = .06				
	σ_e,z→x_ = 10				
	c_e,x→z_ = .2				
	σ_e,x→z_ = 3				
Hebbian Layers					
	*τ_build_* = 4500	*σ_e,x_* = 1		*σ_e,y_* = 3	
	*τ_decay_* = 50000	*σ_e,z_* = 1		*σ_e,y_* = 3	
	*c_e_* = .8				

Figure 3 shows a simulation of the model at key points in time in the experimental task. On the first familiarization trial, an irregularly shaped yellow polygon (binoculars) is presented on the left side of the table. These features are encoded and bound by the model, forming peaks of activation on the left side of the feature-space fields (red hot spots in A, B). Note that the specific feature values cued are somewhat arbitrary; for simplicity, we assume all inputs are distinctive along the shape and color dimensions (see [21]). Once peaks form in the feature-space fields, they project activation into the label-feature fields at the associated feature values (light blue horizontal ridges in C, D). Because no labels are presented (i.e., no labels were provided by the experimenter), the model does not associate the yellow binoculars with a particular name. Comparable events happen on familiarization trial 2. Now, peaks of activation form on the right side of the feature-space fields, binding the curvy, pink features of the spring together. Note the light blue trace on the left side of the feature-space fields: this is the Hebbian trace of the plastic binoculars created on the previous trial.

At the start of the naming event, the experimenter's actions (look into left bucket/point at left location) create a ridge of spatial activation on the left that is propagated across the feature-space fields (E, F). At the same time, the experimenter says the label “Modi!” which propagates a ridge of activation across the label-feature fields at the 7^th^ label position (G, H). As neural interactions grow stronger in the feature-space fields, peaks emerge at the feature values associated with the binoculars—the model recalls that the binoculars were on the left (I, J). This, in turn, sends activation into the feature-label fields which stimulates neurons already stimulated by the presentation of the label. Consequently, a peak emerges at the 7^th^ label position at neural sites associated with the binocular features (K, L). This binds the name “modi” to the binocular features, and a Hebbian process creates a trace of this association that can subserve performance on later trials.

On the test trial, the novel name is presented, passing a ridge of activation into the label-feature fields as before. In addition, the two objects are presented in the task space at new locations (on a tray in the center, bright dots in M, N). As can be seen in panels O and P, the re-presentation of the same label (“get the modi!”) enables the model to recall the features of the binoculars. This passes activation into the feature-space fields, biasing the model to build peaks at the binocular shape and the yellow color. Note that spatial coupling plays a role here, ensuring the multiple features of the same object are correctly bound together. At the end of this simulation, the model correctly ‘picks’ the binoculars.

We used this model to simulate Experiments 1–4, event by event, all with the same parameter setting. To simulate the No-Switch condition of Experiment 1, for example, we presented the model the same sequence of events we gave the child—binoculars on the left, spring on the right, binoculars left, spring right, two buckets and a name, spring on the right, binoculars on the left, then both objects in the center and the label (see Materials and Methods). The model correctly bound the visual features of the objects during the familiarization trials, and it formed associations between visual features and the label when a word was presented. On the test trial, we read out which peak in the feature-space fields was sustained at the end of the trial—this indicated the model's choice. We did this for 100 simulations of each task from Experiments 1–4, and replicated these simulation experiments 12 times to probe the model's robustness. This was akin to running each child through 100 iterations of each task to robustly estimate how that child would respond (in a perfect world where the child would tolerate such a thing), and collecting data on 12 children (the smallest *N* across experiments). Note that the multiple iterations were necessary given the stochastic nature of neural activation in the model (i.e., the neural dynamics were influenced by multiple noise sources from timestep to timestep; see Model and Simulation Details below).

As is clear in Figure 2, the model superbly captured children's behavior across all variants of the Baldwin task. The white bars show average performance of the model across the 12 runs for each experiment. The error bars show the standard deviation of the model's performance relative to each empirical mean across the 12 batches of simulations for each condition. This provides a measure of the model's variability relative to the target empirical value. In all cases, the empirical means are quantitatively near or within the range of variability produced by the model. Moreover, the model standard deviations are well within the range defined by the empirical standard deviations.

### Test of Model

Space is critical both in the model and in contemporary understanding of the neural processes that underlie visual attention and action as well as the binding of features into integrated object representations [6], [32], [37], [38]. Indeed, recent studies show that position dependence persists throughout the ventral visual pathway, even into areas such as the inferior temporal cortex which was once thought to be spatially invariant [39]–[42]. This contrasts with approaches that treat space as a generic featural cue and pursue other binding mechanisms (e.g., neuronal synchrony, see [43], [44]). To examine the special role of space in the model, we asked whether the model was able to map a name to an object if some feature *other than* space was aligned across initial interactions with the objects and the naming event. Thus, during familiarization, two objects were presented on differently colored trays that were always centered, that is, there was a unique color associated with each object but space was always the same (see Fig. 1). During the naming event, no objects were presented, but one of the colors associated with one object was. This was followed by two re-presentation trials during which each object and its appropriate color cue were presented to the feature-space fields. As can be seen in Figure 2, the model failed to use this non-spatial association to pick the correct object at test, performing at chance levels.

Experiment 5 tested this prediction with children. During familiarization, each object was presented at the center of the table but consistently on either a red or a blue tray (see Fig. 1). During the naming event, one of the two colored trays was presented at the center of the table and the experimenter pointed to it saying the label. The objects were then re-presented, one at a time, on their respective trays in the center of the table. The final test event was exactly as in the prior experiments. As can be seen in Figure 2, children picked the object that corresponded with the color presented during naming only .47 of the time, a rate that is not significantly different from chance performance, *t*(15) = −.522, *ns*. Importantly, children were able to match the novel objects to their previously-paired colors; correctly recalling the color when presented with the objects on .70 of memory-check trials that occurred after the main test phase.

One could argue that the lack of binding shown by children in this experiment could be because the act of pointing to the empty colored tray was not viewed by the child as a referential act. However, in Experiment 2 children *succeeded* in binding the name and object when naming occurred in a very similar context—pointing to an empty spatial location. Thus, we argue, the differential binding results across Experiments 2 and 5 confirm the prediction that space—and the spatial direction of attention—is indeed special in its ability to bind names to objects in this task.

### Behavioral Extension

Experiments 1–5 show that children use spatial consistency to bind names to objects in a classic task designed to invite children to read social cues provided by the experimenter. The final question we ask is whether the spatially-grounded processes reflected in these experiments and in the DNF model play a role in everyday social interactions in which toddlers learn object names. These interactions often involve multiple objects in a single context, and those objects are regularly moved about. Nonetheless, some degree of spatial consistency might spontaneously emerge and promote learning.

**Figure 4:**
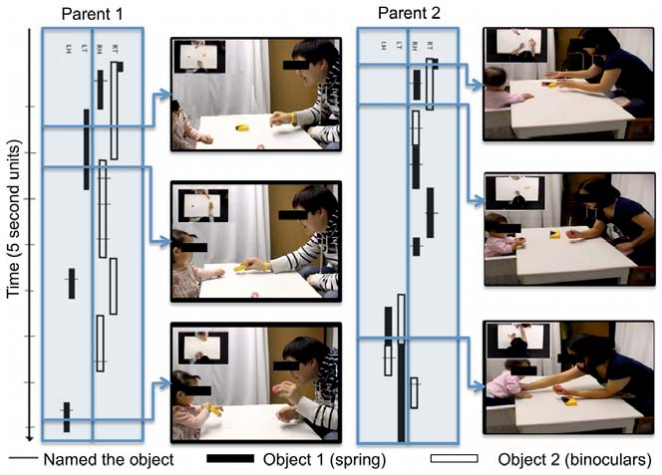
Representative data from two parent-child pairs in Experiment 6. Blue blocks show the time course of the object positions over a 45 second section of the interaction (starting at the top of the figure). Black bars refer to object 1 (binoculars in these examples), white bars refer to object 2 (spring in these examples). Hash marks across bars indicate naming. Right-left spatial position of the object is coded from the child's perspective as in the parent's hand on the left (LH), in the parent's hand on the right (RH), on the table to the left (LT) or on the table to the right (RT). Screen shots from recordings are provided to illustrate the placement of objects at the point of the interaction indicated by the arrow. Insets in pictures are from the overhead cameras. As can be clearly seen, parent 1 kept the objects clearly separated—the binoculars (black bars) are kept on the child's left and the spring (white bars) is on the right. In contrast, parent 2 did not maintain a consistent spatial segregation of the objects. Rather, early in this segment (top of blue block) she kept both objects on the child's left. Later (bottom of blue block) she switched both objects to the child's right side (bottom of figure). Data from the comprehension test reveal that children of parents who kept the objects segregated, like parent 1, learned the words best (see main text).

**Figure 5:**
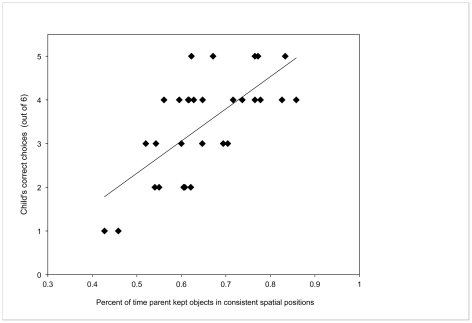
Correlation between parents' spatial consistency and children's learning of novel names in Experiment 6. Graph plots individual children's total number of correct choices across the 6 trials of the novel name learning test according to the spatial consistency ratio of that child's parent. Spatial consistency was defined as the percent of time the parent held the novel objects in the same right-left position (each object relative to the other), out of the total amount of time the objects were in separate hands.

To investigate this, in a sixth experiment we asked caregivers to teach their 17- to 22-month-old children names of two objects novel to the children. Parents were not told the experimental hypothesis nor was the use of space mentioned in any way. An experimenter later tested the children to determine whether they had learned the object names. Videotapes of parent-child interactions were coded to determine the spatial consistency of the objects while on the table, in the parent's hands, or in the child's hands, and to record all naming events. Overall, parents spontaneously maintained a consistent spatial arrangement of the two objects during the social interaction, consistently holding the objects in different hands .75 of the time (i.e., the binoculars in the left hand, the spring in the right hand), and maintaining spatial consistency .65 of the time even when only one object was being presented (e.g., holding and presenting the spring always with the right hand). Interestingly, parents differed in the degree to which they maintained this spatial consistency: the proportion of time during which the two objects were in two different hands ranged from .32–1.0 and the spatial consistency ranged from .43–.86 (see Fig. 4 for data from two caregiver-child pairs). Critically, this mattered for children's learning of the object names: the more consistent a parent kept the spatial location of the objects, the better her child did on the later comprehension task, *r* = .69, *p*<.001, two-tailed (see Fig. 5). Children's learning was not correlated with age (*r* = .23, *ns*), total number of times parents named the objects (*r* = −.21, *ns*), or amount of time parents spent holding the objects (*r* = −.047, *ns*). Moreover, children's own behaviors on the objects did not show a consistent spatial pattern as they most often held objects in both hands and at midline.

In summary, spatial consistency on the part of the mature social partner appears to play a role in naturalistic parent-child interactions and to support word learning. According to our model, such consistency enables children to bind heard names with seen objects over multiple naming events. But why would parents spontaneously maintain this spatial consistency? One likely answer is that parents have the same cognitive system as their children, one that integrates and indexes information in working memory via visuo-spatial attention, and this organization is reflected in social exchanges with children. It is also possible that over the course of prior interactions parents have picked up on the usefulness of spatial consistency and have learned to use it to facilitate communication. Either way, this final experiment highlights the role space plays in grounding children's learning of novel names in real-world social situations.

## Discussion

There is no denying that language is a fundamentally social phenomenon. It is the medium of human communication and thus requires a social connection between those engaged in the process of passing information back and forth. It is not surprising, then, that even young infants tune into the social nature of language and begin to use social cues to meaning and intention in the service of language learning from an early age (for review see [45]). In fact, the contrast between young children's precocious “mindreading” skills and those of other animals [2] as well as atypically developing children [46] has put social cues at the center of explanations of language learning [1]–[4].

The present results and model provide a fine-grained, neurally-grounded, and mechanistic account for children's abilities inspired by the growing number of studies of toddlers and adults showing an exquisite coupling of the body's momentary spatial orientation and internal cognitive operations [8], [47]. Specifically we have shown that children can use consistency in spatial location to bind a novel name to a novel object in an ambiguous naming situation. In this way then, these studies suggest that the direction of visual attention can be used as a deictic reference to bind objects in the physical environment to cognitive variables that can be used in mental operations. Likewise, in the social context of early word learning, the spatially consistent actions by the mature partner align the direction of visual attention and the object that is the momentary “topic” of interest for the two social partners, and in this way may also influence the internal cognitive operations of their social partner [8], [48]. Thus the impressive “mindreading” skills infants demonstrate in the service of word learning need not depend solely on inferential processes or internal models of others' minds, processes which might well be too slow to account for the smoothness of social interactions and adjustments that happen on the order of milliseconds [49], [50]. Instead, shared sensory-motor coupling might emerge as the driving force, a consequence of the shared physical space in which bodies and cognitive systems are embedded.

For these young learners, spatial consistency mattered more than the clarity of the referential intent of the speaker during the naming event. This need not imply that words are not *referential* for young children (see [51] for the importance of this issue in the literature on early lexical development). Words refer in the sense that they are symbols that point. Often this pointing is to a physical object located space; sometimes the point is to an entity in memory that may also be indexed by its experienced location in the world [9], [52]. Within the human cognitive system, then, the referential nature of words may be inherently spatial; pointing to spatially localized objects (or events) in the world or spatially indexed entities in memory. Spatial consistency thus may provide better cues for “reference” in this indexical pointing sense then activities of the speaker at the moment of naming. We suggest that the theoretical construct of reference in early word learning has its mechanistic reality in the literal sense of pointing. Consistent with this idea, recent studies of eye-tracking in adults document the strong influence of words on the direction of visual attention, as adults appear to mandatorily attend to a named object even if it is irrelevant to the task [53] or the location [54] where the named object had been. Fereirra et al. [52] suggest that these word-localized attention effects reflect the fundamental nature of online word comprehension: words automatically direct looking to the location (or remembered location) of a mentioned object (see also, [55], [56]), that is, they literally point to locations for attention.

The adult studies on the strong link between words and the direction of visual attention suggest that the role of spatial consistency in word learning observed here may not be specific to young children but may also be measurable in adults (albeit most likely when limitations are imposed on adult cognitive resources). However, there are also possibilities of change, as the evidence suggests developmental improvement in the ability to update an objects' location with respect to self, given movement by the perceiver [57]–[60]. In the present report, we used a dynamic neural field model that encoded and remembered locations in a table-centered reference frame [33]. Although this is consistent with the spatial cognitive literature, a head- or body-centered frame would have yielded the same results given that children were not actively moved during the procedure. Clearly, however, there are developmental changes in children's ability to stably remember locations in a world-centered frame beyond 18 months (see, e.g., [61]). We suspect such changes may alter how children ground objects and words in space—and how well they may be able to update their memories for those object locations given moving objects or self-movement. Future work will be needed to probe how changing abilities in spatial representations in early childhood may influence the role of spatial consistency in early word learning.

In conclusion, the present experiments and DNF simulations provide new insights into the neural mechanisms that enable the early social coupling children demonstrate in service of word learning. Since Posner's [50] classic paper on attention as a spatial spotlight, considerable evidence shows how visuo-spatial processes are essential for the perception, representation, and tracking of objects. The current findings show these processes at work in early word learning via shared space. The central message of these experiments is that sharing space may be *the* social process that enables the emergence of so-called ‘mindreading’ in early development. What we have done here is to make this process open to analysis, not just at a behavioral level, but also at a neural level via our DNF model. In so doing, we have grounded social word learning in known processes of object recognition and visual binding. This then opens the door to understanding—and experimentally testing—the *mechanisms* that underlie the social and spatially-grounded nature of early word learning.

## Materials and Methods

### Behavioral Studies

#### Ethics Statement

Parents of all child participants provided informed consent prior to the experiment. All experimental protocols were approved by the Indiana University Institutional Review Board. In particular, the committee approved the consent materials which were signed by the parents of the participating children and are on file at the University.

#### Samples

Twenty-four 17–18-month-old children, 12 females, participated in Experiment 1. They were randomly assigned to two between-subject conditions, Switch and No-Switch. All children completed the warm-up trials. Two additional children were replaced due to shyness. Sixteen 17–19-month-old children, 8 females, participated in Experiment 2. All children completed the warm-up trials. Sixteen 17–19-month-old children, 8 females, participated in Experiment 3. Two children were replaced because of fussiness and failure to do the warm-up task. Twenty-eight 17–19-month-old children, 14 females, participated in Experiment 4. Children were randomly assigned to the experimental or control conditions. Three children were replaced due to fussiness or failure to do the warm-up trials. Sixteen 17–18-month-old children, 8 females, participated in Experiment 5. All children completed the warm-up trials. Thirty 17–22-month-old children and their parents, participated in Experiment 6. All children completed the task. Children in all experiments were from monolingual middle-class homes in a Midwestern town. Participant names were obtained from public birth records. They were recruited via a letter sent to their parents and a follow-up phone call.

#### Stimuli

Novel stimuli for all experiments were drawn from a set of five objects: a colorful spring, collapsible binoculars, a noise maker, a transparent cube with moving colored beads inside, and a toy hand drill. Each object was between 5–7 cm in length, 4–7 cm in height, and 4 cm in width. Two identical grey plastic buckets, 15 cm high with a diameter of 12 cm, were used in Experiment 1. Prototypical examples of categories well-known by children this age were used as warm-up and filler items (e.g., cat, ball, duck) all approximately the same size as target items. A small transparent container (10×10 cm, 5 cm high) was used to present objects during testing. This test container was small enough that the objects were virtually on top of each other but both see-able (see Fig. 1). In Experiment 5, two 40 cm diameter colored trays, one red one blue, were used.

#### Procedure and Equipment

In Experiment 1, the child and the experimenter sat across from each other at a small table. The experiment began with warm-up trials designed to familiarize the child with the testing procedure used at the end of the session. The experimenter first introduced the child to a stuffed bear (who will “ask” the test questions). She then presented the child with two familiar objects (e.g., airplane and rabbit), and told the child the names (“See this airplane. Look, here is a bunny”). The experimenter then put the objects in the test container and told the child that he needed to get the object the bear asked for. The bear then “looked” at the container, and said “Get the airplane.” Correct choices were cheered and incorrect choices were corrected. This was repeated with different toys until the child correctly indicated the requested object on 4 out of 4 trials.

The familiarization phase for block 1 then began (see Fig. 1). The target object was presented first, either 25 cm to the right or 25 cm to the left of midline. The experimenter held the object up, saying “Look at this. See this.” for 5 s and then gave the object to the child, by pushing it toward the child following an imaginary line approximately 25 cm off midline so that the child looked and reached to the object on that side. After the child examined the object for approximately 5 s, the experimenter took it back, again moving it along the same imaginary line along which it had been presented. The experimenter then presented the distractor object 25 cm off midline *on the opposite side* and repeated the procedure. This whole procedure was repeated for a total of four familiarization trials. In the No-Switch condition, the target was consistently presented on one side of the table and the distractor on the other. In the Switch condition, the side on which the target and distractor were presented switched from trial to trial. This is the only difference between the two conditions.

Following familiarization, the experimenter placed the target and distracter objects in separate buckets, out of view of the child. The buckets were placed on the table, one 25 cm to the right of midline, the other 25 cm to the left of midline, such that the bucket containing the target was on the same side as the last presentation of the target during the familiarization phase. Next the experimenter tapped one bucket, and said “modi” (or “dawnoo”) three times, while looking straight into the child's eyes. Note that this is a change from Baldwin's [1] original procedure in which the experimenter looked into the bucket. We made this change because in later experiments the experimenter would be looking at an empty location in space (Experiment 1) or to her snapping fingers (Experiment 3). Thus, in order to keep the procedure for our studies consistent, the experimenter looked into the child's eyes during the naming portion of Experiments 1–5. Note that one could argue this change makes the naming event less salient in our experiments compared to Baldwin's. However, in that case the change would be working against our hypothesis. Further, the fact that we replicate her results in the No-Switch condition of Experiment 1 almost exactly, suggests that not looking at the child rather than in the bucket did not alter performance.

Following the naming event, the experimenter took the distracter from the un-named bucket and gave it to the child to examine for approximately 5 s before taking it back. Then the experimenter took the target from the named bucket, gave it to the child for approximately 5 s and took it back. The test phase followed immediately. These trials were structured identically to the warm-up trials but without feedback. Both objects were placed in the test container at midline, and the bear “asked” the child to “Get the modi (or dawnoo).” During this period, the experimenter maintained her gaze directly at the child's eyes. Four test trials with the target and distractor were alternated with 4 filler trials in which the bear asked children to select between pairs of familiar toys previously seen in the warm-up. These were included to maintain interest, to break up the 4 requests for the target and ensure children understood the task. This whole procedure (excluding warm-up) was then repeated with the other target-distractor pair. The order of target-distractor pairs, assignment of objects to target/distractor status, side associated with the naming event and whether the modi or dawnoo set was first or second, were all counter-balanced across children within each condition. The procedure took approximately 25 minutes. During the procedure one video-camera was focused on the child (and parent who typically sat next to or behind the child). A second video camera was focused on the experimenter.

The procedure for Experiment 2 was identical to the No-Switch condition of Experiment 1 with the exception that no buckets were used. During the naming phase all objects were removed so there was just the empty tabletop. The experimenter, while looking in the child's eyes, pointed to the spot on the table where the target object had been introduced during familiarization (that is, 25 cm to the right or left of midline) and said “modi” three times. During the re-presentation phase, the experimenter presented the distractor object (the one associated with the un-named side) first, then the target object (the one associated with the named side). Testing was the same as in Experiment 1. In this experiment, children only received one block of trials due to the oddity of the naming event and because of the consistency of responding in the No-Switch condition of Experiment 1.

The procedure for Experiment 3 was identical to Experiment 2 with the exception of the naming phase during which the experimenter held her arm out at shoulder height to the side the target had been presented on and snapped her fingers while saying the name “modi” three times, all the while looking in the child's eyes.

The procedure for the Space v. Time condition of Experiment 4 was identical to No-Switch condition of Experiment 1 with three exceptions. First, there were 8 total familiarization trials (rather than 4). Second, during the naming event, the experimenter put one of the objects on the table—*at the location associated with the other object*—and with the object in view, pointed to and named it, saying the name, “modi,” three times. Third, there was no re-presentation phase. Instead a 5 s delay was imposed before the experimenter started the first test trial. Children in the Control condition of this experiment participated only from the naming event forward.

The procedure for Experiment 5 was like that of Experiment 1 with the exception that the object-location correspondences were replaced with object-tray-color correspondences. During familiarization, one object was presented twice at midline on a red tray and the other was presented twice at midline on a blue tray. Then, during naming the experimenter brought out one tray with nothing on it and, while looking straight in child's eyes, said “modi” three times. The red tray was used as the target tray for half the children and the blue tray for the other half. The target tray color remained the same on both blocks. During re-presentation, each object was presented on its appropriate tray. The test phase was identical to previous experiments. A memory test followed during which children were asked to put each object on the tray on which it belonged. Performance was greater than .70 across children on these trials.

In Experiment 6 parents were told to play with the two training toys, to show children how they worked and to attempt to teach them their names. They were told the task was about how children attend to objects when learning about new objects and their names and that the parents should be as natural as possible. The experimenter left the room after turning on the cameras. Two cameras were used, one facing the child directly and one overhead. Parents demonstrated the training toys to their child for 2 minutes. Then the experimenter came in and played with the child with a filler toy for approximately 2 minutes. This was done to familiarize the child with the experimenter prior to the test trials. The experimenter left and the parent demonstrated the experimental toys for another 2 minutes. The experimenter then tested the child in the name comprehension test. On each trial, two choices were given to the child to handle. They were then taken by the experimenter and placed on the two ends of an 46 cm tray. Holding the tray close to her body, with the two objects fully in view by the child but unreachable, the experimenter asked the child to get one object by name (e.g., “Where is the modi? get the modi,”). The tray was pushed forward for the child to indicate their choice. The two experimental objects served as the choice objects on 6 trials with each object the labeled target on 3 trials. The remaining 6 trials used three filler sets, asking for each object once. The side of the correct choice was randomly determined for each trial.

#### Coding and Analysis

Children's responses during the test trials of all experiments were coded for the object selected on each trial by a scorer who was blind to the procedure and who also did not see the familiarization and re-presentation trials and so did not know the right answer. The first point, touch, or a pick of an object was scored as the child's choice. A second coder scored the experimenter's direction of gaze and the parent's behavior during testing to ensure that that neither influenced the child's choices. A third coder re-scored 25% of these tapes; reliability exceeded 90% on all categories for all experiments. In addition, for Experiment 6, the moment-by-moment interaction of the mother and child as they played with the objects was coded using MacShapa coding software. At each moment of the session a primary coder recorded the location of the objects in terms of the right-center-left placement on the table or in the parent or child's hand. Naming events by the parent and child were also recorded and tagged to the moment in time when they occurred. A second coder coded 15% of the data. Agreement was over 87% for all participants and all codes.

### Model and Simulation Details

Below we define the equations for the four dynamic neural fields in the model used to capture children's early word-learning performance: the space-shape field (ssf), the space-color field (scf), the label-shape field (lsf), and the label-color field (lcf). Each field equation specifies the rate of change of neural activation, 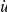, over two field dimensions. We adopt the following convention for the dimensions: *x* refers to the spatial dimension, *y_1_* and *y_2_* specify the two visual feature dimensions (shape, color), and *z* refers to the label dimension.

Activation in the space-shape field, *u_ssf_*, is governed by the following equation:
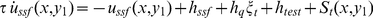
(1)

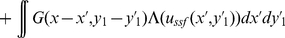


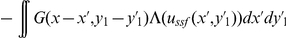
(2)

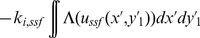
(3)

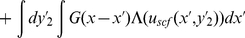
(4)

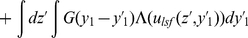
(5)

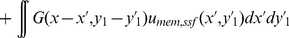
(6)

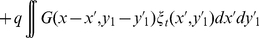
(7)where 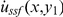 is the rate of change of the activation level for each bi-modal neuron across the spatial dimension, *x*, and the shape dimension, *y_1_*, as a function of time, *t*. The constant 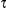 sets the time scale of the dynamics. The current activation in the field is given by, 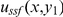. This component is negative so that activation changes in the direction of the neuronal resting level, 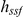. The next term in the equation specifies noise on the neuronal resting level, where 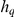 is the strength of the noise modulation, and 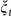 is a white noise process sampled independently through time from a uniform distribution. The final two terms in line (1) specify task-specific contributions (see below): 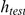 lowers the resting level on the test trials, increasing competition via stronger inhibition, while *S_t_(x,y_1_)* specifies localized inputs to the field caused by, for instance, the presentation of the toy on the left or right side of the table. Note that inputs to the field generally took the form of localized, two-dimensional Gaussian distributions (see (8) below), except where noted in the model procedures section below.

The next term in the equation (line 2) specifies locally-excitatory interactions within the space-shape field. These excitatory interactions are given by the convolution of a two-dimensional Gaussian kernel with a sigmoidal threshold function. The Gaussian kernel was specified generically by:
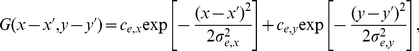
(8)with excitatory strengths, *c_e_*, and excitatory widths, *σ_e_*. The level of activation required to enter into the interaction was determined by the following generic sigmoidal function:
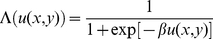
(9)where *β* is the slope of the sigmoid. The slope determines whether neurons close to threshold (i.e., 0) contribute to the activation dynamics with lower slope values permitting graded activation near threshold to influence performance, and higher slope values ensuring that only above-threshold activation contributes to the activation dynamics.

Line 3 in the equation specifies local contributions to lateral or surround inhibition. This local component of the inhibitory interactions is specified by the convolution of a Gaussian kernel with a sigmoidal function. Note that the widths of the lateral inhibitory interactions, *σ_i_*, are larger than the excitatory widths, *σ_e_*. In addition to lateral inhibition, there is also a global inhibitory contribution that is constant across the field dimensions and scaled by a strength parameter, *k_i_*.

The fourth contribution to the field dynamics specifies the contribution of above-threshold activation in the space-color field (scf) to the space-shape field (ssf). All above-threshold activation in the space-color field is integrated across the color feature dimension, *y_2_*, and projected uniformly across the shape dimension in the space-shape field. Note that this projection is via the convolution of a Gaussian kernel with the integrated activity. This enables the ‘binding’ of multiple features across the shared spatial dimension. For instance, a peak in the space-color field will project a spatial ‘ridge’ across the space-shape field at the spatial location associated with the space-color peak. In a sense, the space-color peak reaches out to find an associated spatial pattern in the space-shape field. A similar form of coupling happens in line 5 between the space-shape field (ssf) and the label-shape field (lsf). In this case, above-threshold activation in the label-shape field is integrated across the label dimension and projected (via a Gaussian convolution) uniformly across the space dimension in the space-shape field. This enables peaks with particular shape values to reach out and find associated shape patterns in the space-shape field.

The sixth contribution to the field dynamics reflects a Hebbian contribution. Here, traces in a Hebbian layer (see (10) below) are convolved with a Gaussian kernel and passed as input to the space-shape field. This boosts activation in the space-shape field at the sites of previously consolidated peaks, priming the field dynamics to re-build peaks at these same locations. The final contribution to the activation dynamics in line 7 is spatially correlated noise which is the convolution of a Gaussian kernel with a field of white noise sources scaled by the noise strength parameter, *q*.

The equation for activation in the space-color field, *u_scf_*, is identical to the equation for the space-shape field except the feature dimensions, *y_1_* and *y_2_*, are reversed. Next, the equation for activation in the label-shape field, *u_lsf_*, is identical to the equation for the space-shape field except the feature dimensions, *x* and *z*, are reversed. Finally, the equation for the label-color field, *u_lcf_*, is identical to the equation for the space-shape field except the feature dimensions, *y_1_* and *y_2_*, are reversed and the feature dimensions, *x* and *z*, are reversed. Note that in the Faubel and Schöner [24] model, the label dimension consisted of a collection of one-dimensional fields to capture the arbitrary ordering of labels. We realized a similar picture with a continuous label dimension, *z*, by ensuring that the labels probed were always metrically far from one another and using parameters that instantiated winner-take-all competion between labels.

Longer-term learning in the DNF model is achieved by a Hebbian layer associated with each neural field. Each layer specifies learning via an activation trace variable, *u_mem_*, as follows (example shown is for the space-shape Hebbian layer):
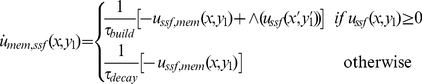
(10)This specifies that the rate of change of activation traces in the Hebbian layer is determined by a build timescale, *τ_build_*, if there is above threshold activation in the space-shape field (i.e., activation in that field ≥0). In this case, traces grow toward a value of 1. Otherwise, Hebbian traces decay back to 0 according to a decay timescale, *τ_decay_*. Note that the timescale of change in the Hebbian layers is much longer than the real-time scale of the neural activation dynamics (i.e., *τ_build_*≫*τ*).

### Model parameters

Table 1 reports the global field parameters, the parameters for the space-feature and label-feature fields, the parameters for the Gaussian projections between fields, and the parameters for the Hebbian layers. To set the specific parameter values, we adopted the following procedure. First, we began with parameter values from a related project probing the neural dynamics of early word learning. The goal of this complementary modeling project—which used the same neural architecture—was to examine whether a single model of early word learning could qualitatively capture a host of phenomena, including enhanced performance in a comprehension task relative to a production task early in word learning; the developmental emergence of a shape-bias when generalizing novel names for novel solid objects [62]; referent-selection in a canonical “fast-mapping” task; and learning of names at different levels of a category hierarchy. This model qualitatively captured all of these phenomena.

Next, we modified values of several parameters from this initial parameter set with an eye toward quantitatively simulating the empirical means from the present study across all experiments with a single parameter set (with the exception of changes in inputs needed to capture the experimental procedures—see below). This led to changes in six parameter values (relative to those in [24]). First, we increased the resting level of the space-feature fields from *h* = −6.8 to −6.35 (see Table 1) and increased the strength of the projections across the spatial dimension from *c_e_* = .15 to .162. This enhanced the excitatory interactions within the space-feature fields as well as across the spatial dimension, eliminating the feature-binding errors that occured on some trials. Next, we lowered the resting level of the label-feature fields from *h* = −6.5 to −11.55 to implement the winner-take-all interactions from Faubel and Schöner [24]. Third, we lowered the resting levels of the fields during the test trials by −0.1 units. This increased competitive interactions in the fields at test, similar to the increased competition on forced-choice trials used by Samuelson et al. [21]. Next, we decreased the strength of spatially-corrleated noise in the fields from *q* = .1 to .075. Finally, we decreased the build timescale (*τ_build_*) in the Hebbian layers from 5000 to 4500 to accumulate long-term memory traces a bit faster. This was particularly critical for robust performance in the control condition of Experiment 4 where the model had to quickly learn about an object in a single trial.

In summary, then, the core parameter values shown in Table 1 reflect a constrained simulation approach in that only 13.6% of the parameter values were modified relative to the initial parameter set imported from a different modeling project with different goals. Although we did have to tailor six parameter values to meet our objectives, it is useful to place these parameter modifications in context. In the present study, we simulated children's performance across 28 total test trials from 7 different conditions (see Fig. 2). Moreover, we required that the model correctly bind features and locations across 30 total familiarization trials from 7 conditions. Thus, in total, the model had to correctly bind object features and quantitatively match children's performance across a total of 58 trials.

In addition to the parameter values in Table 1, we also had to specify the details of the inputs used across experimental conditions. The labeing input had strength *c_z_* = 8 and width *σ_z_* = 1. The pointing input had strength *c_x_* = 4 and width *σ_x_* = 1 in Experiments 1 and 2 and strength *c_x_* = 1.9 and width *σ_x_* = 40 in Experiment 3 to capture the broad spatial cue in that experiment. Note that there was no pointing input in Experiments 4 and 5 (as in the procedure with children). Finally, the object input had strenght *c* = 5 and width *σ* = 3. White noise sampled independently through time from a uniform distribution was added to the strength of the object inputs.

The object inputs were always presented at spatial positions 20 and 70 during familiarization and re-presentation, except in Experiment 5 where the objects were presented at the center of the field (position 45). Which object was assigned to each spatial position was determined by the experimental procedure (see main text). During test, the object presented at position 70 was moved to central position 40, and the object presented at position 20 was moved to central position 50; thus, if the model had a spatial bias at test it would consistently choose the incorrect object (which did not occur). Note also that the spatial position of the pointing input in Experiment 3 was shifted to position 15 instead of position 20. This reflected the poorly localized spatial input in this experiment.

### Simulations

Simulations were conducted in MATLAB (Mathworks Inc.) on a HP Pavillion e9820t computer with 8 processors running at 3.3 gigahertz (code available upon request). The dynamic field equations were integrated using the Euler method. Each run of the model simulated the experience of a single child in an experiment consisting of between 2 and 10 events (see Fig. 1). The same model with the same parameters (see Table 1) was used in all simulation sets. Models were initialized with a small starting vocabulary corresponding that of the 18-month-old children who participated in the experiments. This starting vocabulary was identical to the early vocabulary used in our previous modeling work.

Trials began with the field at the neuronal resting level for 100 timesteps. On each familiarization trial, an object's shape and color were presented to object- and location-specific sites in the space-shape and space-color fields respectively for 300 time steps. During the pointing event on the naming trial, a ridge of activation centered at the spatial location of one of the two objects was sent into the shape-feature fields for 700 timesteps. At the same time, a ridge of activation corresponding to the novel label was presented to the label-feature fields. The two re-presentation events were identical to the familiarization events. The test event began with the presentation of the object features corresponding to the two objects at a central location in each of the feature-space fields for 300 timesteps. Then, after a 300 timestep delay, a ridge of activation corresponding to the name was presented in each of the label-feature fields for 700 timesteps. The response was determined by taking the location of the maximum activation in the feature-space fields at the end of a 100 timestep response interval. Individual simulations verified that the response peaks were in the exact location of one of the test objects.

Simulations for the Switch condition of Experiment1, and Experiments 2–4 were identical except for differences to replicate the experimental details. The objects switched locations in the feature-space fields to capture the Switch condition of Experiment 1. No bucket inputs were presented to the feature-space fields on the naming trial to simulate Experiment 2. To capture the more diffuse spatial cue during the naming event in Experiment 3, the ridge sent in to the feature-space fields was broader and poorly localized (see Table 1). In the Space v. Time condition of Experiment 4, the spring, for example, was presented on the right during familiarization but the left during the naming trial, while in the Control condition only the naming and test events occurred. For Experiment 5 a centrally-located but distinctive color cue was presented along with each object. Otherwise, this simulation proceeded as for Experiment 2. Results are averages over 12 batches of 100 simulations (i.e., 12 participants) in each experiment.

## References

[1] Baldwin DA (1993). Early referential understanding: Infants' ability to recognize referential acts for what they are.. Dev Psychol.

[2] Tomasello M (2007). If they're so good at grammar, then why don't they talk? hints from apes' and humans' use of gestures.. Lang Learn Dev.

[3] Papafragou A (2001). Mindreading and verbal communication.. Mind Lang.

[4] Bloom P (2002). Mindreading, communication and the learning of names for things.. Mind Lang.

[5] Zmigrod S, Berhnhard H (2010). Temporal dynamics of unimodal and multimodal feature binding.. Atten Percept Psychophys.

[6] Johnson JS, Hollingworth A, Luck SJ (2008). The role of attention in the maintenance of feature bindings in visual short-term memory.. J Exp Psychol Hum Percept Perform.

[7] Hayhoe M, Ballard D (2005). Eye movements in natural behavior.. Trends Cogn Sci.

[8] Smith LB (2007). The dynamic lift of developmental process.. Dev Sci.

[9] Richardson DC, Spivey M (2000). Representation, space and hollywood squares: Looking at things that aren't there anymore.. Cognit.

[10] Richardson DC, Kirkham NZ (2004). Multimodal events and moving locations: Eye movements of adults and 6-month-olds reveal dynamic spatial indexing.. JEP:General.

[11] Bloom L (1970). Language development; form and function in emerging grammars.

[12] Brown R, Bellugi U (1964). Three processes in the child's acquisition of syntax.. Harvard Educational Review.

[13] Woodward AL, Hoyne KL (1999). Infants' learning about words and sounds in relation to objects.. Child Dev.

[14] Gallese V, Goldman A (1998). Mirror neurons and the simulation theory of mind-reading.. Trends Cogn Sci.

[15] Gallese V, Gernsbacher MA, Heyes C, Hickok G, Iacoboni M (2011). Mirror neuron forum.. Perspectives on Psychological Science.

[16] Jancke D, Erlhagen W, Dinse HR, Akhavan AC, Giese M (1999). Parametric population representation of retinal location: Neuronal interaction dynamics in cat primary visual cortex.. J Neurosci.

[17] Johnson JS, Spencer JP, Luck SJ, Schöner G (2009). A dynamic neural field model of visual working memory and change detection.. Psychol Sci.

[18] Wang XJ (2001). Synaptic reverberation underlying mnemonic persistent activity.. Trends Neurosci.

[19] Erlhagen W, Schoner G (2002). Dynamic field theory of movement preparation.. Psychol Rev.

[20] Simmering VR (2008). Generalizing the dynamic field theory of spatial cognition across real and developmental time scales.. Brain Res.

[21] Samuelson LK, Schutte AR, Horst JS (2009). The dynamic nature of knowledge: Insights from a dynamic field model of children's novel noun generalizations.. Cognition.

[22] McMurray B, Horst JS, Toscano J, Samuelson LK, Spencer JP, Thomas M, McClelland J (2009). Towards a New Grand Theory of Development? Connectionism and Dynamic Systems Theory Reconsidered.

[23] Mel BW (1997). SEEMORE: Combining color, shape, and texture histogramming in a neurally inspired approach to visual object recognition.. Neural Comput.

[24] Faubel C, Schoner G (2008). Learning to recognize objects on the fly: A neurally based dynamic field approach.. Neural Netw.

[25] Johnson JS, Spencer JP, Schöner G (2009). A layered neural architecture for the consolidation, maintenance, and updating of representations in visual working memory.. Brain Res.

[26] Luck SJ, Girelli M, McDermott MT, Ford MA (1997). Bridging the gap between monkey neurophysiology and human perception: An ambiguity resolution theory of visual selective attention.. Cogn Psychol.

[27] Desimone R, Gross CG (1979). Visual areas in the temporal cortex of the macaque.. Brain Res.

[28] Gross CG, Rocha-Miranda CE, Bender DB (1972). Visual properties of neurons in inferotemporal cortex of the macaque.. J Neurophysiol.

[29] Damasio AR (1989). Time-locked multiregional retroactivation: A systems-level proposal for the neural substrates of recall and recognition.. Cogn.

[30] von der Malsburg C (1999). The what and why of binding: The modeler's perspective.. Neuron.

[31] Riesenhuber M, Poggio T (1999). Are cortical models really bound by the “Binding Problem”?. Neuron.

[32] Treisman AM, Gelade G (1980). A feature-integration theory of attention.. Cogn Psychol.

[33] Lipinski J, Schneegans S, Sandamirskaya Y, Spencer JP, Schöner G (2011). A neurobehavioral model of flexible spatial language behaviors.. J Exp Psychol Learn.

[34] Lipinski J, Spencer JP, Samuelson LK (2010). Biased feedback in spatial recall yields a violation of delta rule learning.. Psychon B and Rev.

[35] Samuelson LK (2002). Statistical regularities in vocabulary guide language acquisition in connectionist models and 15–20-month-olds.. Dev Psychol.

[36] Regier T (2005). The emergence of words: Attentional learning in form and meaning.. Cognitive Sci.

[37] Wolfe JM (1994). Guided search 2.0: A revised model of visual search.. Psychon Bull Rev.

[38] Larsson J, Heeger DJ (2006). Two retinotopic visual areas in human lateral occipital cortex.. J Neurosci.

[39] Op De Beeck H, Vogels R (2000). Spatial sensitivity of macaque inferior temporal neurons.. J Comp Neurol.

[40] DiCarlo JJ, Maunsell JHR (2003). Anterior inferotemporal neurons of monkeys engaged in object recognition can be highly sensitive to object retinal position.. J Neurophysiol.

[41] Aggelopoulos NC, Rolls ET (2005). Scene perception: Inferior temporal cortex neurons encode the positions of different objects in the scene.. Eur J Neurosci.

[42] Kravitz DJ, Vinson LD, Baker CI (2008). How position dependent is visual object recognition?. Trends Cogn Sci (Regul Ed).

[43] Singer W (1999). Neuronal synchrony: A versatile code for the definition of relations?. Neuron.

[44] Singer W, Chalupa LM, Werner JS (2004). Synchrony, oscillations and relational codes.. The visual neurosciences (2nd ed.).

[45] Deak GO, Flom RA, Pick AD (2000). Effects of gesture and target on 12- and 18-month-olds' joint visual attention to objects in front of or behind them.. Dev Psychol.

[46] Sigman M, Ruskin E (1999). Continuity and change in the social competence of children with autism, down syndrome, and developmental delays.. Monogr Soc Res Child Dev.

[47] Richardson DC (2007). The art of conversation is coordination.. Psychol Sci.

[48] Shockley K, Santana M, Fowler CA (2003). Mutual interpersonal postural constraints are involved in cooperative conversation.. J Exp Psychol Hum Percept Perform.

[49] Pereira AF (2008). Social coordination in toddler's word learning: Interacting systems of perception and action.. Connect Sci.

[50] Posner MI (1980). Orienting of attention.. Q J Exp Psychol.

[51] Waxman SR, Gelman SA (2009). Early word-learning entails reference, not merely associations.. Trends Cogn Sci (Regul Ed).

[52] Ferreira F, Apel J, Henderson JM (2008). Taking a new look at looking at nothing.. Trends Cogn Sci (Regul Ed).

[53] Salverda AP, Altmann GTM (in press). Attentional capture of objects referred to by spoken language.. J Exp Psychol Hum Percept Perform.

[54] Estes Z, Verges M, Barsalou LW (2008). Head up, foot down: Object words orient attention to the objects' typical location.. Psychol Sci.

[55] Altmann GTM (2004). Language-mediated eye movements in the absence of a visual world: The ‘blank screen paradigm’.. Cognition.

[56] Knoeferle P, Crocker MW (2007). The influence of recent scene events on spoken comprehension: Evidence from eye movements.. J Mem Lang.

[57] Newcombe N, Huttenlocher J, Learmonth A (1999). Infants' coding of location in continuous space.. Infant Behav Dev.

[58] Huttenlocher J, Newcombe N, Sandberg EH (1994). The coding of spatial location in young children.. Cognit Psychol.

[59] Bushnell EW, McKenzie BE, Lawrence DA, Connell S (1995). The spatial coding strategies of one-year-old infants in a locomotor search task.. Child Dev.

[60] Newcombe N, Huttenlocher J, Drummey AB, Wiley JG (1998). The development of spatial location coding: Place learning and dead reckoning in the second and third years.. Cognitive Dev.

[61] Schutte AR, Spencer JP (2009). Tests of the dynamic field theory and the spatial precision hypothesis: Capturing a qualitative developmental transition in spatial working memory.. J Exp Psychol Hum Percept Perform.

[62] Landau B, Smith L, Jones S (1988). The importance of Shape in early lexical learning.. Cognit Dev.

